# Smokers’ Affective Responses to COVID-19-Related Health Warnings on Cigarette Packets: The Influence of Delay Discounting

**DOI:** 10.1093/ntr/ntab176

**Published:** 2021-09-01

**Authors:** Chris R H Brown, Paul Faulkner

**Affiliations:** Department of Psychology, University of Roehampton, London, UK; Department of Psychology, University of Roehampton, London, UK

## Abstract

**Introduction:**

The addition of graphic health warnings to cigarette packets can facilitate smoking cessation, primarily through their ability to elicit a negative affective response. Smoking has been linked to COVID-19 mortality, thus making it likely to elicit a strong affective response in smokers. COVID-19-related health warnings (C19HW) may therefore enhance graphic health warnings compared to traditional health warnings (THW). Further, because impulsivity influences smoking behaviors, we also examined whether these affective responses were associated with delay discounting.

**Methods:**

In a between-subjects design, 240 smokers rated the valence and arousal elicited by tobacco packaging that contained either a C19HW or THW (both referring to death). Participants also completed questionnaires to quantify delay discounting, and attitudes towards COVID-19 and smoking (eg, health risks, motivation to quit).

**Results:**

There were no differences between the two health warning types on either valence or arousal, nor any secondary outcome variables. There was, however, a significant interaction between health warning type and delay discounting on arousal ratings. Specifically, in smokers who exhibit low delay discounting, C19HWs elicited significantly greater subjective arousal rating than did THWs, whereas there was no significant effect of health warning type on arousal in smokers who exhibited high delay discounting.

**Conclusion:**

The results suggest that in smokers who exhibit low impulsivity (but not high impulsivity) C19HWs may be more arousing than THWs. Future work is required to explore the long-term utility of C19HWs, and to identify the specific mechanism by which delay discounting moderates the efficacy of tobacco health warnings.

**Implications:**

The study is the first to explore the impact of COVID-19-related health warnings on cigarette packaging. The results suggest that COVID-19-related warnings elicit a similar level of negative emotional arousal, relative to traditional warnings. However, COVID-19 warnings, specifically, elicit especially strong emotional responses in less impulsive smokers, who report low delay discounting. Therefore, there is preliminary evidence supporting COVID-19 related warnings for tobacco products to aid smoking cessation. Additionally, there is novel evidence that, for some warnings, high impulsiveness may be a factor in reduced warning efficacy, which may explain poorer cessation success in this population.

Despite the implementation of public health interventions, roughly 34.1 million US adults and approximately 6.9 million British adults continue to smoke.^1,2^ To counter this ongoing public health problem tobacco health warnings have been added to cigarette packets. These written and pictorial warnings about specific smoking-related health risks (eg, cancer, tooth damage, emphysema) significantly increase negative emotional reactions to cigarettes, increase the motivation to quit, and reduce smoking behaviors.^3^ However, there is a need to refine these interventions to optimize their impact on smoking cessation.

There is a potential opportunity, therefore, to harness the COVID-19 pandemic in this regard, by including COVID-19-specific health information in health warnings on cigarette packets. Recent large-scale meta-analyses and large sample population studies suggest that smokers are more likely to experience severe health outcomes after contracting COVID-19. For example, compared to never-smokers, both current and former smokers are more likely to be hospitalized after contracting COVID-19, have poorer in-hospital outcomes, and are more likely to die from COVID-19.^4–8^

Further, evidence from several large sample surveys suggest that the pandemic may also be influencing smokers’ attitudes towards cessation. For instance, comparing United Kingdom data from the first month of the first national lockdown (March-April, 2020) to data from the past 5 years suggests that, despite increases in factors that promote smoking (ie, alcohol consumption, psychological distress), cigarettes smoked per day actually decreased during the first UK lockdown.^9^ Similar reductions in smoking were also found in some smokers in other surveys conducted across the UK, US, Italy, India, and South Africa.^10,11^ This reduction in smoking behaviors appears to be driven by changes in the intention to quit, as self-reported motivation to quit and the number of reported quit attempts also increased during the first UK lockdown.^12,13^ The evidence, therefore, suggests that COVID-19-related health concerns may be a suitable target to enhance the efficacy of cigarette packet graphic health warnings to promote smoking cessation.

Currently, only one study has explored reactions to health messages highlighting the elevated COVID-19 severity for smokers.^14^ In this study, participants viewed traditional health warnings (THW) and COVID-19-related health warnings (C19HW) as social media posts. Based on self-reported perceived effectiveness, negative emotion, and perceived harm, the authors reported that C19HW messages were no more or less effective than THW messages. Importantly, the messages presented by Grummon et al. were text-based and did not include any imagery. As such, it is currently unknown whether combined written and pictorial COVID-19-related information can enhance graphic health warnings on cigarette packets.

Evidence suggests that the primary mechanism by which THWs increase both the intention to quit and the initiation of a quit attempt is by eliciting a negative affective response.^15–18^ The current investigation, therefore, sought to compare the ability of C19HWs with the ability of THWs to elicit self-reported affective arousal and negative valence. It was predicted that ratings of affective arousal and negative valence would be greater for a group of smokers who rated images of cigarette packets with C19HWs relative to those who rated packets with THWs. Further, based on the increase in motivation to quit smoking during the pandemic,^9–13^ we explored whether the Fear of COVID-19 (as measured by the Fear of Coronavirus scale)^19^ moderated the impact of C19HWs on subjective arousal and valence.

In order to detect whether the C19HWs could also influence other outcome variables, we measured the secondary dependent variables of motivation to quit and perceived probability of negative health outcomes and perceived severity of negative health outcomes, including both symptoms unique to smoking (eg, cancer), symptoms linked to COVID-19 (eg, respiratory failure), and COVID-19 itself. Increases in the motivation to quit and increased perceptions of health risks after exposure to tobacco health warnings have been found to be mediated through the magnitude of emotional response to the warnings,^18^ thus placing them as more secondary variables of interest. Though the primary-secondary variable distinction was implied rather than explicit within out pre-registration (see Supplementary Material 1 for pre-registered correlations between affective ratings and secondary variables). We also expected these secondary dependent variables to be higher in the C19HW condition as a result of the hypothesized stronger negative emotional response to these warnings.

Based on evidence that individual differences in the level of nicotine dependence and impulsivity influence both smoking behaviors and the ability to quit smoking,^20–25^ we also examined their moderating effect on affective responses to C19HWs on cigarette packets in exploratory moderation analyses. It was predicted that affective responses would be lowest in smokers who exhibit the highest levels of nicotine dependence and the steepest delay discounting.

## Methodology

Prior to data collection, the sample size, hypotheses, materials, and analyses were pre-registered on the Open Science Framework (OSF; Pre-registration link: https://osf.io/fzx8e).

### Participants

The pre-registered target sample was 240 participants (2 groups of 120), derived from an *a piori* power analysis (*d* = .42, α = .05, β = .90; based on previous meta-analysis.^3^ Pre-registered inclusion criteria required that participants be UK residents, aged 18 or over, and currently identify as a smoker. Equal numbers of male and female participants were also recruited within each group. In a deviation from the registered inclusion criteria, all participants were included regardless of whether they recalled the precise text on the smoking warning accurately, as the open-ended recall answers were often ambiguous (data available via the OSF: osf.io/qp2n9). Participants were recruited from Prolific Academic online recruitment pool in exchange for financial payment (£2.50).^26^ Recruitment occurred between September 28^th^ and October 5^th^, 2020. Inclusion criteria were initially filtered through the Prolific Academic pre-screen responses, but were confirmed with responses in the survey.

In the final sample, 119 participants were randomly assigned to the C19HW group, and 121 were randomly assigned to the THW group. All participants responded to identical survey links and were unaware of the alternative group condition. Participant characteristics are reported in Table 1. Consistent with our pre-registered recruitment plan, exclusions occurred for incomplete submissions (*n* = 1) and for those who no longer identified as a smoker (*n* = 15). Inconsistent with our pre-registered recruitment plan, three participants reported being neither male nor female. All three were randomly assigned to the THW group, therefore for purely statistical reasons they were not included in the final sample. Additionally, one additional male participant was allocated to the THW condition rather than the C19HW condition.

**Table 1.** Participant Characteristics. Unless Otherwise Stated, Values Denote Means, and Values in Brackets Denote Standard Deviations. THW = Traditional Health Warning; C19HW = COVID-19 Health Warning; LT = lifetime; SJWS = Shiffman-Jarvik Withdrawal Scale; FTND = Fagerström Test for Nicotine Dependence; DDT = Delay Discounting Task. Mean Hours Since Last Cigarette Variable was Biased by Infrequent Smokers Responses, Thus we Calculated the Proportion of Smokers who had Smoked in the Past 2, 12 and 24 Hours to Present the Distribution of Data. COVID-19 Risk Pre-Knowledge Refers to the Proportion of Participants who Reported Having Knowledge of the Link Between COVID-19 Mortality and Smoking Prior to the Survey. Significance was Computed Using Between Subjects T-Tests or Chi-Squared Tests. Bayes Factors were Computed Using Non-Directional Two-Sided Between-Subjects Comparison with Default Prior (Cauchy = .707). Bayes Factors Below .33 Denote Sensitive Data Showing Evidence for the Null

	THW group (n = 121)	C19HW group (n = 119)	Cohen’s d	p-value	Bayes factor
Age	35.15 (12.10)	36.50 (12.50)	.11	.394	.20
Gender (*M/F*)	61/60	59/60	-	.999	.16
Cigarettes per day	10.96 (7.39)	10.98 (7.52)	.02	.856	.14
Start age	17.81 (5.44)	17.10 (3.10)	–.16	.216	.29
LT Quit attempts	3.96 (3.01)	4.75 (4.50)	–.21	.112	.47
Hours since last cigarette	83.75 (661.68)	36.63 (223.48)	–.01	.462	.18
Smoked in past 2 h (*smoked/total*)	82/121	72/119	-	.241	.30
Smoked in past 12 h (*smoked/total*)	99/121	92/119	-	.386	.19
Smoked in past 24 h (*smoked/total*)	108/121	104/119	-	.653	.11
Electronic cigarette user (*user/total*)	35/121	36/119	-	.822	.15
SJWS	3.20 (.64)	3.26 (.75)	.07	.510	.16
FTND	3.19 (2.38)	3.27 (2.44)	.04	.780	.15
COVID-19 risk pre- knowledge	94/121	89/119	–	.598	.16
DDT *k-*value	.04 (.05)	.04 (.05)	–.02	.907	.14

### Materials and Procedure

Data was collected online using Qualtrics via the Prolific Academic online recruitment pool.^26^ Participants first completed a demographic questionnaire (developed in-house), after which they completed a smoking history questionnaire (developed in-house) to quantify current smoking status, hours since smoking, average cigarettes smoked per day (and for how long they smoked this amount), the nicotine products they used (eg, rolled cigarettes, vape/electronic cigarettes), age of smoking initiation, and lifetime attempts to quit. Participants then completed the 6-item Fagerstrom Test for Nicotine Dependence (FTND),^27^ and the 25-item Shiffman-Jarvik withdrawal scale,^28^ which quantified symptoms of tobacco withdrawal along 5 subscales (cigarette craving, psychological withdrawal symptoms, physical withdrawal symptoms, sedation, and appetite), all of which can be summed to create a total score.

Participants were then exposed to the experimental manipulation, in which one group rated six current UK cigarette packet images with a non-COVID-19-related death text warning, whilst the other group rated six cigarette packets with a COVID-19-related death text warning. The C19HW contained the text label: “*Smoking increases the risk of death from Covid-19*”; whilst the THW contained the text label: “*Smoking increases the risk of early death”.* The warnings were, therefore, matched on the severity of the outcome, sentence structure, and framing of the risk. The six graphic images presented on the packets were identical across groups, and depicted two instances of medical patients on a mechanical ventilator, one patient on oxygen, one undergoing defibrillation, a person coughing, and a dead person in a body bag. These were selected for their relevance to both traditional, non-COVID-19-related, and COVID-19-related death warnings. All images are available via the OSF: osf.io/qp2n9.

Ratings were along dimensions of valence and affective arousal measured with a Self-Assessment Manikin (SAM) for external reference.^29^ The responses were along 9-point scales ranging from 1 (“extremely unpleasant”) to 9 (“extremely pleasant”) for valence, and 1 (“calm/bored”) to 9 (“excited/agitated”) for arousal. Alongside the scale, the SAM appeared as a simple pictorial depiction of each level of valence and arousal. To increase exposure to the warnings, cigarette packets were presented for 4-seconds before the rating scales appeared.

After the affective ratings, participants completed the single-item Motivation to Stop Smoking Scale (MTSS).^30^ The MTSS ranges from 1 (“I don’t want to stop smoking”) to 7 (“I REALLY want to stop smoking and intend to in the next month”).

The perceived probability and severity of smoking-related health outcomes were then recorded based on recommendations by Kaufman et al.^31^ Participants rated how likely/probable they were to personally experience a range of 11 negative health outcomes along a 7-point scale (“very low possibility” to “very high possibility”), after which they reported how severe they expected the outcomes to be if they contracted each condition on a 5-point scale (“not at all severe” to “extremely severe”). The average probability and severity was recorded for symptoms unique to smoking and not linked to COVID-19 (ie, reproductive/sexual dysfunction, tooth damage, lung cancer, throat cancer, premature aging, emphysema; Cronbach’s alphas: probability = .88; severity = .76), and those strongly linked to COVID-19 (ie, respiratory illness, increased susceptibility to illness, weakened immune system; Cronbach’s alphas: probability = .87 and severity = .86). Two items (ie, stroke, heart disease) were excluded from both totals for their less well-known (at time of recruitment) link to COVID-19.

Participants then completed COVID-19-related measures, specifically, the belief of COVID-19 contagion (ie, perceived COVID-19 infection probability; 4-items) and belief of COVID-19 consequences scales (ie, perceived COVID-19 severity; 4-items).^32^ Ratings were along continuous scales with the slider ranging from 0 to 100. This was followed by the potential moderator of Fear of COVID-19/coronavirus scale (7-items),^19^ in which responses range along a 5-point scale (“strongly disagree” to “strongly agree”). Participants then reported their prior awareness of the smoking-COVID-19 mortality link before the study (“yes”/“no”), and also typed out briefly what the text warning on the cigarette packet had been, to assess memory for the warnings.

Finally, participants completed the 27-item delay discounting monetary choice questionnaire,^33^ which requires participants to choose between two hypothetical options; one of which provides a smaller financial reward after a short delay (eg, £20 today) and the other of which provides a larger financial reward deterministically awarded after a longer delay (eg, £24 in 80 days). The amount of money and temporal delays were varied to provide a profile of delay discounting reported as a *k-*value.

A cigarette monetary value measure was pre-registered and presented after the health warnings, but due to survey instruction error unreliable responses were recorded. This measure and data are not reported here, but are available through the OSF: osf.io/qp2n9).

### Statistical Analyses

The pre-registered primary analysis was a between-groups comparison of arousal and valence, though group differences in secondary factors were also compared. This analysis was performed using both frequentist and Bayesian independent samples *t*-tests. To follow-up, exploratory moderating factors were included in hierarchical regression analyses, modeling the independent effects and interaction between predictor variables at different steps. The variables identified as possible moderating factors were financial delay discounting, fear of COVID-19, and nicotine dependence. To delineate any significant interaction between groups and potential predictors, unregistered follow-up simple slopes analysis was conducted using the *processR* package in R.^34^ Pre-registered Bayesian correlations were also computed within each group, regardless of significance, to assess the evidence for or against within-group relationships.

In follow-up regression analyses, covariates were also included to determine whether significant results found were independent of potential confounds, specifically, gender, education, and age (Supplementary Material 2). In independent regression analyses, these covariates were also entered as possible moderators in the model to explore their effects (Supplementary Material 4).

In an addition to the pre-registered analysis, for the regression analysis, bootstrapped 95% confidence intervals were computed with 5000 resamples to account for violations of normality.^35^ All continuous variables were standardized for regression analyses to allow bootstrapping of standardized *β*s. To account for unexpected outliers within the regression predictor variables, we also excluded cases if they were of over 2SDs from the mean. Inclusion or exclusion of outliers did not alter the significance of the key results (*p* < .05). All analyses were conducted using R and JASP software, which allows the computation of Bayes factors (BF).^36^ These were calculated for all direct between-group comparisons and all within-group correlations. A directional one-tailed default prior (Cauchy = .707) was utilized for between-groups comparisons, due to the lack of previous knowledge.^37^ The prior predicted effect size for Bayesian correlations was a two-tailed stretched beta prior of 1, the default within JASP. Bayes factors allow the inference of the magnitude of evidence for both the null (BF < .33) and experimental effect (BF > 3), with a BF between .33 and 3 signifying inconclusive data.^38,39^

## Results

### Group Comparisons

The main pre-registered comparison, reported in Table 2, revealed no significant difference between groups in terms of either arousal, valence, or any secondary outcome variables. Further, Bayes Factors all showed evidence favoring the null hypothesis.

**Table 2.** Group Comparisons for Each Dependent Variable. Values Denote Means (Standard Deviations in Brackets). THW = Traditional Health Warning; C19HW = COVID-19 Health Warning. Significance was Computed Using Between-Subjects T-Tests. Bayes Factors were Computed Using Directional Between-Subjects Comparison with Default Prior (Cauchy = .707). Bayes Factors Below .33 Denote Sensitive Data Showing Evidence for the Null

		THW group (n = 121)	C19HW group (n = 119)	Cohen’s d	p-value	Bayes factor
Primary dependent variables	Arousal	4.01 (1.66)	4.12 (1.68)	.06	.621	.22
	Valence	3.18 (1.23)	3.13 (1.13)	.05	.729	.11
Secondary dependent variables	Motivation to quit	3.61 (1.79)	3.40 (1.68)	-.12	.355	.08
	COVID-19 specific health - probability	14.07 (4.04)	13.68 (4.16)	-.10	.457	.09
	Smoking specific health - probability	27.60 (7.74)	27.54 (6.83)	-.01	.945	.13
	COVID-19 specific health - severity	11.47 (2.56)	11.71 (2.62)	.09	.483	.27
	Smoking specific health - severity	22.35 (4.30)	22.50 (4.46)	.04	.782	.18
	Belief of COVID-19 contagion	52.74 (22.53)	48.98 (23.40)	-.16	.207	.07
	Belief of COVID-19 consequences	34.45 (17.40)	35.41 (18.83)	.05	.682	.20

### Moderation Effects

To explore whether there was a difference between groups on arousal and valence ratings when moderated by individual differences in delay discounting, level of nicotine dependence, or fear of COVID-19, we modeled the interaction between warning type and each of these three variables on both arousal and valence using separate hierarchical linear regressions. Almost all participants rated the packets as having a negative valence (97% valence rating < 5), revealing that arousal ratings reflected negative emotional response.

#### Delay Discounting

**Figure 1.**
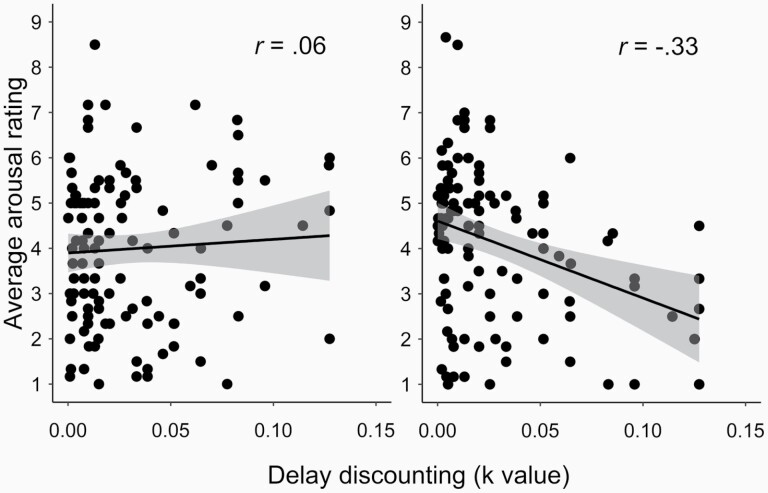
Relationships between average arousal rating of cigarette packets and average *k*-value in the monetary choice delay discounting task for Tradition Health Warning (left) and COVID-19 Health Warning (right). Higher values on delay discounting reflect preference for smaller short-term reward. Error bands reflect standard error. Correlation coefficients are presented for each group. The difference between immediate and delayed reward can be divided into small (eg, £3), medium (eg, £12), and large (eg, £28) amounts. The relationship between delay discounting and arousal ratings was consistently significant across small, medium, and large value differences, in the C19HW group (*p* < .01); *r*_*small*_: –.35; *r*_*medium*_: –.25; *r*_*large*_ = –.26. Whilst in the THW group the effect was consistently non-significant (*p* > .136), *r*_*small*_: .08; *r*_*medium*_= .07; *r*_*large*_ = .14.

The addition of the delay discounting *k-*value (19 outliers > 2 SD excluded) revealed a significant interaction between the type of health warning and delay discounting (*R*^*2*^ = .06, *F*(3,221) = 4.17, *p* = .005), the full analysis is reported in Table 3. The effect was driven by a significant negative relationship between arousal and *k*-values in the C19HW group, *r(108)* = –.33, *p* < .001, BF_10_ = 62.65, but a non-significant relationship within the THW group, *r(113)* = .06, *p* = .541, BF_10_ = .14. Scatterplots depicting this relationship appear in Figure 1.

**Table 3.** Hierarchical Regressions Including Independent Main Effects Of Health Warning Type and Delay Discounting on Arousal (Step1), The Interaction Between Health Warning Type and Delay Discounting on Arousal (Step 2). All Continuous Variables were Standardised (ie, Z-Score). Step 1: *R*^*2*^ = .02, *F*(2,222) = 2.02, *P* = .135; Step 2: *R*^*2*^ = .06, *F*(3,221) = 4.17, *P* = .005; Bootstrapped 95% Confidence Intervals (CI) were Calculated with 5000 Resamples, a Lower-Upper Bound Interval Non-Inclusive of Zero Denotes a Significant Result. Further Inclusion of Age, Gender, Education, and Current Withdrawal State as Exploratory Covariates did not Account for the Significant Interaction (See Supplementary Material 2)

		β	t	p-value	95% CI lower bound	95% CI upper bound
Step 1: Independent effects	Health warning type	.04	.57	.567	–.18	.34
	Delay discounting	–.14	2.06	.041	–.27	–.01
Step 2: Interaction	Health warning type	.06	.58	.561	–.18	.33
	Delay discounting	.08	.63	.530	–.12	.24
	Health warning × delay discounting	–.39	2.97	.003	–.65	–.13

To explore the relationship between arousal ratings at different levels of delay discounting across both groups, a follow-up simple slopes analysis was conducted on the standardized data. The analysis revealed significantly higher arousal ratings for the C19HWs, versus THWs, in participants who exhibited low delay discounting (–1SD), *β* = .42, *t*(221) = 2.41, *p* = .017, 95% CI_bootstrapped_[.08, .76], and a non-significant effect of health warning-type on arousal ratings in participants who exhibited high delay discounting (+1SD), *β* = –.31, *t*(221) = –1.69, *p* = .092, 95% CI_bootstrapped_[–.68, .05]. The pattern of results gave the appearance of a cross-over interaction (see Supplementary Material 3 for plot), in which C19HWs elevated arousal ratings for low impulsive individuals more so than impulsive individuals experienced attenuated arousal.

Repeating the hierarchical regression with valence as the outcome variable revealed no significant effects or interactions, *R*^*2*^ < .01, *p* > .706. The relationship between delay discounting and valence ratings was non-significant in both groups, *r* < .09, *p* > .354, BF_10_ < .18, with Bayes factors strongly favouring the null.

#### Fear of COVID-19

A hierarchical regression analysis with fear of COVID-19 (4 outliers > 2 SDs excluded) and health warning type revealed a significant predictive model, *R*^2^ = .09, *F*(2,233) = 10.89, *p* < .001. There was no difference between the health warning types, *β* = .05, *t*(233) = .36, *p* = .720, 95% CI_bootstrapped_[–.21, .30], but a significant positive relationship emerged between fear of COVID-19 and arousal, *β* = .29, *t*(233) = 4.64, *p* < .001, 95% CI_bootstrapped_[.17, .42]. The addition of the interaction yielded no significant change in the model, *R*^2^_*change*_ < .01, *F*(1,232) = .11, *p* = .746. The overall relationship between fear of COVID-19 and arousal was driven by positive correlations in both groups, C19HW: *r(115)* = .28, *p* < .001, BF_10_ = 11.23, and THW: *r(117)* = .30, *p* < .001, BF_10_ = 30.87. Thus, even without exposure to COVID-19 warnings, fear of COVID-19 was a significant predictor of self-reported arousal when viewing the cigarette packets.

Repetition of the analysis with valence as the outcome variable revealed non-significant predictive models for both the independent effects, *R*^*2*^ = .02, *F*(2,223) = 2.49*, p* = .085, and the interaction, *R*^*2*^ = .02, *F*(2,223) = 1.65*, p* = .178. In both groups, fear of COVID-19 only weakly correlated with valence, *r <* –.15, *p* > .101, BF < .44.

Further regression analyses with nicotine dependence, as well as demographic factors (ie, age, gender, education), as moderating variables yielded no significant interactions with warning type (all *p’s* > .403; see Supplementary Material 4), though age-predicted increased arousal ratings across both groups.

## Discussion

In the current investigation, we explored whether the inclusion of text C19HWs, versus THWs, on cigarette packets resulted in higher subjective negative affective responses to graphic health warnings in smokers. Our results indicate that, in the whole group, C19HWs were perceived as equally negative and affectively arousing as THWs. Importantly, however, our results also showed that low impulsive individuals experienced heightened arousal when viewing C19HWs, relative to THWs.

The fact that including COVID-19-related warnings on cigarette packets did not substantially increase negative affective ratings across all smokers is consistent with other recent findings, specifically that smokers rated social media messages that contained THWs and C19HWs as equally negative (ie, fear, anxiety, sadness).^14^ Therefore, despite the recent evidence that COVID-19 has prompted an increase in the motivation to quit,^12^ the current evidence suggests that C19HWs may not be more effective than THWs in eliciting a negative affective response, and subsequent motivation to quit, when ignoring the influence of impulsivity on participants’ responses.

Our investigation is the first to show that the affective reaction to some cigarette health warnings is moderated by impulsive reward-based decision making (ie, delay discounting). This finding appears consistent with a general delay discounting trait across both financial and health outcomes.^40^ That is, those who are more focused on future financial outcomes may also be more cognizant of potential future health outcomes, and have a stronger affective reaction when considering the possibility of a potential COVID-19 infection. Alternatively, findings from the reward processing literature have found that individuals who score low on self-reported reward-seeking behavior are more emotionally reactive to aversively conditioned stimuli.^41,42^ Based on these findings, impulsive reward prioritization may compete with, and suppress, affective reactions to a salient COVID-19 threat.

Impulsive decision-making has been found to be a strong predictive factor for the continuation of smoking and relapse. Specifically, individuals who are successful in quitting smoking having greater disposition to select delayed larger rewards than current smokers, and the impulsive preference for short-term reward is a strong predictive factor for relapse.^43,44^ The current results, therefore, point to a potential reason for why impulsive smokers may persist with smoking despite the introduction of health warnings.

Interestingly, there was no difference between the groups on arousal when moderated by fear of COVID-19. Although it was expected that smokers who were fearful of COVID-19 would be especially sensitive to C19HWs, these smokers may already have formed the association between the outcomes depicted on the graphic health warnings (eg, lung damage, death) and COVID-19. The pictorial depiction of these outcomes alone may therefore elicit a strong affective response in COVID-19 fearful smokers, even without the COVID-19 related text. This interpretation is consistent with the fear of COVID-19 positively correlating with arousal ratings in both the THW and C19HW groups.

Against our prediction, the findings were limited to arousal ratings. Though unexpected, this result is consistent with previous research which found manipulations of health warning salience correlated with arousal ratings but not valence.^45^ Importantly, arousal is the affective dimension most strongly implicated in changes in motivation to quit smoking.^18^

While there was no difference in the arousal ratings induced by C19HWs and THWs when ignoring the influence of delay discounting, C19HWs were shown to produce equal affective ratings to the THWs. This suggests that C19HWs do have potential as an additional warning message alongside THWs, perhaps when implemented to reduce message habituation. In future, the utility of C19HWs is likely to be dependent on the progression of the virus. If successful intervention reduces the prevalence of COVID-19 to that of a less common seasonal condition,^46^ the affective impact of the COVID-19-specific warnings may change. Future research is therefore needed to determine the ability of C19HWs to influence smoking behaviors in the months following the COVID-19 pandemic.

In addition, public health may benefit from research that attempts to identify which health warnings are effective at eliciting a strong emotional response in highly impulsive smokers, with a view towards aiding cessation in this subgroup who are notoriously less likely to successfully quit smoking.^23,47^ Though previous research has highlighted the need for variation across messages,^48^ and explored the efficacy of different warnings across demographic differences,^49^ until now no study has explicitly examined whether individual differences in impulsivity influence responses to smoking-related health warnings.

In the current investigation, the focus was on the affective reaction in current smokers who may experience increased motivation to quit during COVID-19.^12^ We are however unable to generalize to other groups such as former smokers, who may be at risk of relapsing during the pandemic.^50^ Though unable to broadly elicit an elevated effective response in all current smokers, C19HWs may be more effective in preventing relapse in former smokers. Future studies may wish to examine this hypothesis.

Future research is also required to determine whether the affective ratings for other negative smoking outcomes are moderated by delay discounting. We selected a single “early death” THW, which matched the severity, framing, and wording of the C19HW. We were, therefore, unable to assess whether delay discounting moderates the impact of health warnings for other conditions, such as cancer, on negative affective arousal.

It should be noted that the novel moderating effect of delay discounting was discovered through exploratory analyses without statistical correction, and though consistent with the extant literature on impulsivity, and Bayesian analysis revealing “very strong evidence” for the experimental hypothesis (BF > 30),^39,44^ future research should aim to replicate and build on this specific exploratory finding.

Our results suggest that C19HWs on cigarette packets may elicit equal emotional responses to THWs, or even enhance affective responses in low impulsive smokers. Therefore, these novel warnings may have some efficacy in aiding smoking cessation, though future work is required to elucidate real-world impact, especially after or during the latter stages of the COVID-19 pandemic. The current investigation is the first to identify delay discounting as a moderator of health warning effectiveness, and may provide one explanation as to why impulsive individuals continue to smoke despite the presentation of health warnings.

## Supplementary Material

A Contributorship Form detailing each author’s specific involvement with this content, as well as any supplementary data, are available online at https://academic.oup.com/ntr.

ntab176_suppl_Supplementary_Materials_S1

ntab176_suppl_Supplementary_Materials_S2

ntab176_suppl_Supplementary_Materials_S3

ntab176_suppl_Supplementary_Materials_S4

ntab176_suppl_Supplementary_Taxonomy_Form

## Data Availability

The raw and processed datasets are freely available for download via the Open Science Framework (OSF). Open access link: https://osf.io/qp2n9.
