# Supplementary material for: Smokers’ Affective Responses to COVID-19-Related Health Warnings on Cigarette Packets: The Influence of Delay Discounting
Source: Nicotine Tob Res. 2021 Sep 1;25(2):221–7. doi: 10.1093/ntr/ntab176 (PMC8499830; doi:10.1093/ntr/ntab176)
Supplement: ntab176_suppl_Supplementary_Materials_S1 [file ntab176_suppl_supplementary_materials_s1.docx]

**SUPPLEMENTARY MATERIALS 1**

**Pre-registered correlation analyses**

One of the preregistered (<https://osf.io/fzx8e>) secondary hypotheses was that the magnitude of the negative affective ratings to both traditional health warnings (THW) and COVID19 related health warnings (C19HW) on packets would correlate with secondary variables linked to successful smoking cessation. That is, arousal would be positively correlated with motivation to quit smoking, perceived severity and probability of negative health outcomes linked uniquely to smoking (“smoking symptoms”) such as cancer or tooth damage, and those linked to both smoking and COVID19 (“COVID symptoms”) such as respiratory illness and weakened immune system. It was expected that valence would be negatively correlated with these variables, reflecting more negative ratings. See Table S1.1 for full results.

|  |  | Motivation to quit | Probability - smoking symptoms | Probability - COVID symptoms | Severity - smoking symptoms | Severity - COVID symptoms |
| --- | --- | --- | --- | --- | --- | --- |
| Arousal | *r* | .10 | .11^+^ | .10 | .03 | .13* |
|  | BF_10_ | .24 | .32 | .27 | .09 | .54 |
| Valence | *r* | -.11 | -.10 | -.12^+^ | -.18** | -.19** |
|  | BF_10_ | .31 | .28 | .47 | 3.55 | 6.17 |

Table S1.1. Correlations between affective arousal and valence ratings, and secondary dependent variables. Smoking symptoms refers to negative health outcomes unique to smoking, and COVID symptoms refers to negative health outcomes linked to both smoking and COVID19. Values denote Pearson’s correlation coefficients, with Bayes factors (BF) presented below. Bayes factors were computed using bi-direction beta-stretched prior of 1. Bayes factors below .33 denote sensitive data showing evidence for the null. Significant results are highlighted as thus: ^+^ *p* < .1; * *p* < .05; ** *p* < .001.
