# Supplementary material for: Smokers’ Affective Responses to COVID-19-Related Health Warnings on Cigarette Packets: The Influence of Delay Discounting
Source: Nicotine Tob Res. 2021 Sep 1;25(2):221–7. doi: 10.1093/ntr/ntab176 (PMC8499830; doi:10.1093/ntr/ntab176)
Supplement: ntab176_suppl_Supplementary_Materials_S2 [file ntab176_suppl_supplementary_materials_s2.docx]

**SUPPLEMENTARY MATERIALS 2**

**Covariate analysis**

To assess whether the significant interaction between health warning type, traditional health warning (TWH) versus COVID19-related health warning (C19HW), and delay discounting *k-*value was not due to possible extraneous variables, a follow-up unregistered hierarchical regression analysis was conducted with an additional step for covariates included.

Covariates included for analysis were gender (male/ female), age, education, and nicotine withdrawal state. Education was measured as the highest level of education achieved within UK qualifications along an ordinal scale with six levels: GCSE, A-levels, post-6th form non-degree awards, undergraduate degree, master’s degree, and doctoral/professional degree. Six participants were excluded from the analysis due them preferring not to report their highest level of education. Nicotine withdrawal state was measured as the total score of the Shiffman-Jarvik withdrawal scale (Shiffman-Jarvik, 1976).

The analysis revealed that the initial covariate regression model was significant, with both age and current withdrawal state independently predicting increased arousal (see Table S2.1). Importantly, the interaction between cigarette packet health warning type and delay discounting remained a significant despite the inclusion of covariates.

|  |  | *β* | *t* | p-value | 95% CI lower bound | 95% CI upper bound |
| --- | --- | --- | --- | --- | --- | --- |
| Step 1: Covariates | Gender | -.02 | .14 | .892 | -.27 | .25 |
|  | Age | .14* | 2.10 | .037 | .002 | .28 |
|  | Education | -.04 | .65 | .515 | -.17 | .08 |
|  | Withdrawal state | .25*** | 3.75 | < .001 | .10 | .39 |
| Step 2: Independent effects | Gender | -.07 | .03 | .610 | -.32 | .19 |
|  | Age | .14 | 2.01 | .046 | .002 | .26 |
|  | Education | -.04 | .57 | .571 | -.17 | .09 |
|  | Withdrawal state | .25*** | 3.74 | < .001 | .10 | .40 |
|  | Health warning type | .04 | .30 | .762 | -.23 | .28 |
|  | Delay discounting | -.15* | 2.26 | .025 | -.29 | -.02 |
| Step 3: Interaction | Gender | -.06 | .47 | .641 | -.31 | .19 |
|  | Age | .13 | 1.90 | .059 | -.01 | .26 |
|  | Education | -.05 | .73 | .465 | -.18 | .07 |
|  | Withdrawal state | .24*** | 3.64 | < .001 | .10 | .38 |
|  | Health warning type | .04 | .34 | .734 | -.22 | .29 |
|  | Delay discounting | .02 | .238 | .812 | -.17 | .20 |
|  | Health warning × delay discounting | -.34* | 2.61 | .010 | -.57 | -.08 |

Table S2.1. Hierarchical regression assessing delay discounting and health warning types’ joint effect on subjective arousal ratings, whilst controlling for extraneous variables. Covariates were included in Step 1, independent main effects of health warning type and delay discounting on arousal were included in Step 2, the interaction between health warning type and delay discounting on arousal was assessed at Step 3. All continuous and ordinal variables were standardised (ie, Z-score). Step 1: *R^2^* = .07, *F*(4,212) = 4.17, *p* = .003; Step 2: *R^2^* = .10, *F*(6,211) = 3.69, *p* = .002; Step 3: *R^2^* = .12, *F*(7,211) = 4.22, *p* < .001. Bootstrapped 95% confidence intervals (CI) were calculated with 5000 resamples, a lower-upper bound interval non-inclusive of zero denotes a significant result. Significant results are highlighted as thus: * *p* < .05; ** *p* < .01; *** *p* < .001.
