# Supplementary material for: Smokers’ Affective Responses to COVID-19-Related Health Warnings on Cigarette Packets: The Influence of Delay Discounting
Source: Nicotine Tob Res. 2021 Sep 1;25(2):221–7. doi: 10.1093/ntr/ntab176 (PMC8499830; doi:10.1093/ntr/ntab176)
Supplement: ntab176_suppl_Supplementary_Materials_S3 [file ntab176_suppl_supplementary_materials_s3.docx]

**SUPPLEMENTARY MATERIALS 3**

**Simple slopes moderation analysis**

To investigate the interaction between health warning type and delay discounting on mean arousal rating, we conducted simple slopes analysis assessing the relationship between health warning type and delay discounting at both low (-1SD from the mean) and high (+1SD from the mean) delay discounting *k*-value. The simple slopes plot is presented in Figure S3.1, and was created using *processR* R-package shiny app (https://cardiomoon.shinyapps.io/processR/; based on the PROCESS macro, Hayes, 2012).

**
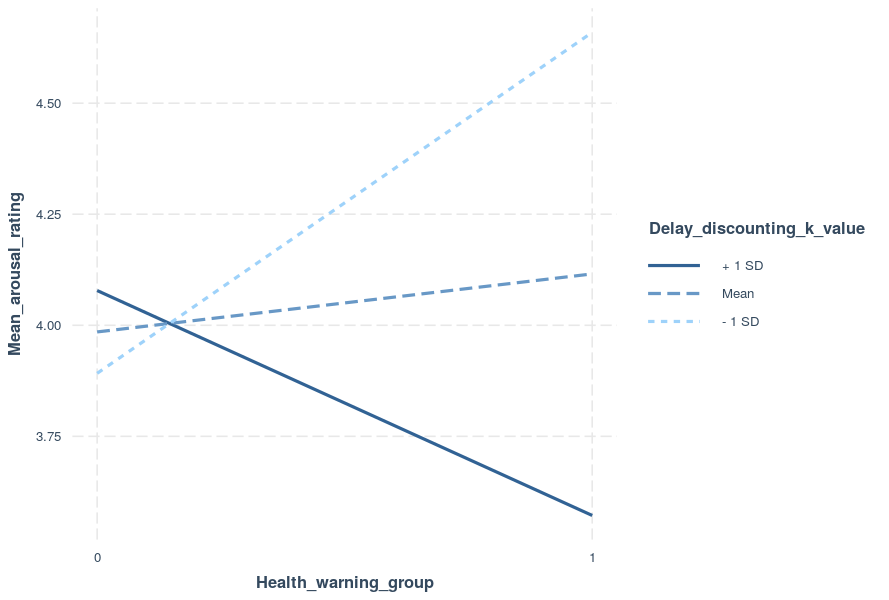
**

Figure S3.1. Simple slopes analysis on unstandardised mean subjective arousal ratings reflecting the interaction between delay discounting and health warning group. The slopes are calculated for high delay discounting (+1SD) and low delay discounting (-1SD). X-axis labels are 0 = Traditional Health Warning; 1 = COVID19 Health Warning.
