# Supplementary material for: Smokers’ Affective Responses to COVID-19-Related Health Warnings on Cigarette Packets: The Influence of Delay Discounting
Source: Nicotine Tob Res. 2021 Sep 1;25(2):221–7. doi: 10.1093/ntr/ntab176 (PMC8499830; doi:10.1093/ntr/ntab176)
Supplement: ntab176_suppl_Supplementary_Materials_S4 [file ntab176_suppl_supplementary_materials_s4.docx]

**SUPPLEMENTARY MATERIALS 4**

**Moderation analyses**

***Nicotine dependence***

To assess the potential interaction between nicotine dependence and health warning type on subjective arousal ratings, the total Fagerstrom test for nicotine dependence (FTND) score was included in the model, as well as its interaction with health warning type (see Table S4.1).

|  |  | *β* | *t* | *p*-value | 95% CI lower bound | 95% CI upper bound |
| --- | --- | --- | --- | --- | --- | --- |
| Step 1: independent effects | Health warning | .06 | .47 | .642 | -.20 | .31 |
|  | Nicotine dependence | .11 | 1.77 | .078 | -.01 | .25 |
| Step 2: Interaction | Health warning | .06 | .47 | .642 | -.19 | .31 |
|  | Nicotine dependence | .06 | .68 | .495 | -.14 | .25 |
|  | Health warning × Nicotine dependence | .10 | .78 | .436 | -.17 | .38 |

Table S4.1. Hierarchical regressions assessing the independent main effects of health warning type and Fagerstrom test for nicotine dependence on arousal (Step1), and the interaction between health warning type and Fagerstrom test for nicotine dependence on arousal (Step 2). All continuous variables were standardised (ie, Z-score). Step 1: *R^2^* = .01, *F*(2,237) = 1.69, *p* = .187; Step 2: *R^2^* = .02, *F*(3,236) = 1.33, *p* = .266. Bootstrapped 95% confidence intervals (CI) were calculated with 5000 resamples, a lower-upper bound interval non-inclusive of zero denotes a significant result.

***Age***

To assess the potential interaction between age and health warning type on subjective arousal ratings, age in years was included in the model, as well as its interaction with health warning type (see Table S4.2). Eleven participants were excluded for being over 2 SD of the mean (Age < 61). Inclusion of these participants did increase the strength of the interaction between age and health warning type, with older participants showing a trend for increased subjective arousal for the traditional health warning type with age. This however failed to reach significance, *β* = -.24, *t* = 1.83, *p* = .068, CI_bootstrapped_[-.49, .02].

|  |  | *β* | *t* | *p*-value | 95% CI lower bound | 95% CI upper bound |
| --- | --- | --- | --- | --- | --- | --- |
| Step 1: independent effects | Health warning type | .10 | .78 | .435 | -.16 | .36 |
|  | Age | .14 | 2.09 | .038 | .01 | .26 |
| Step 2: Interaction | Health warning type | .10 | .78 | .436 | -.15 | .14 |
|  | Age | .21 | 2.24 | .026 | .02 | .37 |
|  | Health warning × age | -.14 | 1.09 | .277 | -.39 | .11 |

Table S4.2. Hierarchical regressions assessing the independent main effects of health warning type and age (in years) on arousal (Step1), and the interaction between health warning type and age on arousal (Step 2). All continuous variables were standardised (i.e. Z-score). Step 1: *R^2^* = .01, *F*(2,237) = 1.69, *p* = .187; Step 2: *R^2^* = .02, *F*(3,236) = 1.33, *p* = .266. Bootstrapped 95% confidence intervals (CI) were calculated with 5000 resamples, a lower-upper bound interval non-inclusive of zero denotes a significant result.

***Gender***

To assess the potential interaction between gender and health warning type on subjective arousal ratings, gender (male/female) was included in the model as a categorical predictor, as well as its interaction with health warning type (see Table S4.3).

|  |  | *β* | *t* | *p*-value | 95% CI lower bound | 95% CI upper bound |
| --- | --- | --- | --- | --- | --- | --- |
| Step 1: independent effects | Health warning type | .03 | .50 | .619 | -.19 | .32 |
|  | Gender | -.03 | .48 | .631 | -.32 | .18 |
| Step 2: Interaction | Health warning type | .09 | .45 | .656 | -.59 | .99 |
|  | Gender | -.01 | .13 | .900 | -.38 | .34 |
|  | Health warning × Gender | -.07 | .31 | .761 | -.60 | .44 |

Table S4.3. Hierarchical regressions assessing the independent main effects of health warning type and gender (male/female) on arousal (Step1), and the interaction between health warning type and gender on arousal (Step 2). Step 1: *R^2^* = .002, *F*(2,237) = .24, *p* = .789; Step 2: *R^2^* = .02, *F*(3,236) = 1.33, *p* = .266. Bootstrapped 95% confidence intervals (CI) were calculated with 5000 resamples, a lower-upper bound interval non-inclusive of zero denotes a significant result.

***Education***

To explore influence of health warning type on arousal ratings when moderated by highest level of education, the independent effect of education and its interaction with health warning type was included in a regression model (see Table S4.4). Highest level of education achieved based on UK qualifications, was measured as an ordinal variable, with six levels: GCSE, A-levels, post-6^th^ form non-degree awards, undergraduate degree, master’s degree, and doctoral/professional degree. Seven participants chose not to report their level of education and were therefore not included in the analysis.

|  |  | *β* | *t* | *p*-value | 95% CI lower bound | 95% CI upper bound |
| --- | --- | --- | --- | --- | --- | --- |
| Step 1: independent effects | Health warning type | .05 | .39 | .697 | -.21 | .31 |
|  | Education | -.01 | -.13 | .898 | -.14 | .12 |
| Step 2: Interaction | Health warning type | .05 | .39 | .696 | -.21 | .31 |
|  | Education | .04 | .42 | .678 | -.14 | .22 |
|  | Health warning × Education | -.10 | .74 | .462 | -.36 | .16 |

Table S4.4. Hierarchical regressions assessing the independent main effects of health warning type and highest level of education on arousal (Step1), and the interaction between health warning type and highest level of education on arousal (Step 2). All ordinal and continuous variables were standardised (ie. Z-score). Step 1: *R^2^* = .001, *F*(2,230) = .08, *p* = .922; Step 2: *R^2^* = .003, *F*(3,229) = .24, *p* = .872. Bootstrapped 95% confidence intervals (CI) were calculated with 5000 resamples, a lower-upper bound interval non-inclusive of zero denotes a significant result.
